# Application of an Automated Digital Image-Processing Method for Quantitative Assessment of Cracking Patterns in a Lime Cement Matrix

**DOI:** 10.3390/s20143859

**Published:** 2020-07-10

**Authors:** Maciej Szeląg

**Affiliations:** Faculty of Civil Engineering and Architecture, Lublin University of Technology, 40 Nadbystrzycka Str., 20-618 Lublin, Poland; maciej.szelag@pollub.pl; Tel.: +48-81-538-4762

**Keywords:** cement matrix, cracks detection, image analysis, cracking pattern, fractal dimension, *ImageJ*

## Abstract

The paper presents an original approach to the localization and analysis of the cracking patterns of cement composites. The lime cement matrix modified with microsilica was evaluated under a two-phase thermal load. For quantitative detection and analysis of thermal cracks, an image-processing method was applied. For this purpose, an original image double-segmentation method was developed using machine-learning algorithms. Among other things, the fractal analysis was used to describe the morphology and the thermal evolution of the cracking patterns. The basic mechanical characteristics were examined and the results indicated a very high correlation between tensile strength and all cracking patterns’ parameters. This allows high-quality estimation of the mechanical properties of the lime cement matrix to be carried out on the basis of measurement and evaluation of morphology of the thermal cracking patterns. Knowledge in this field contributes to the development of non-destructive testing methods in cement composites technology, in terms of localization of and tracking the cracking patterns.

## 1. Introduction

Determination of characteristics of the hardened cement composites is possible due to destructive and non-destructive testing. Determining in a destructive way the parameters of the material embedded in the actual structure requires a violation of its integrity, because the sample to be tested must be taken, e.g., as a borehole. The place of sampling itself must then be supplemented accordingly. Very often taking a cement composite sample for testing from an existing structure is limited or even impossible. Destructive testing in concrete technology allows for precise determination of physical and mechanical properties of the material [1,2]. However, it requires the use of heavy equipment, which by nature is laboratory equipment, e.g., strength machines. In order to determine the characteristics of cement composites and their degree of degradation as a function of service life, non-destructive testing methods are increasingly being sought that allow the material to be tested without compromising its integrity. An example of such testing in the technology of cement composites is the sclerometric method of determining the material strength, using Schmidt’s hammer [3,4,5,6,7]. The operation of Schmidt’s hammer is based on the use of the dynamic method of measurement by evaluating the change of energy of the spring hammer after the reflection from the surface tested. The reflection value is read directly from the scale placed on the Schmidt’s hammer.

In order to assess the degradation of the cement composite, a cracking pattern analysis can also be performed on its surface [8,9,10,11,12,13,14,15,16,17,18]. However, a cracking patterns analysis is problematic for methodological reasons because the simplest localization and tracking methods (e.g., portable measuring microscope for measuring crack opening width) are burdened with a large measurement error related to the human factor. Much more accurate and reliable crack analysis methods are based on a digital analysis of a cracked surface fragment of a cement composite. However, the main problem of methods based on a digital image analysis is the correct separation of cracks from the rest of the material. The most common reason for this is the poor contrast between the cracking patterns and the undamaged part of the cement composite, because the cracks appear as black lines on a grey background. At present, with the progressive development of digital technologies, considerable development of research methods in this area can be observed.

There are many studies in the literature which aim only to extract cracks from the surface of the material as accurately as possible. So far, many analysis techniques have been developed that are more or less accurate, and thus use more or less of the computational resources and time needed for analysis. The simplest and at the same time the quickest and least accurate analysis techniques include methods based on an image thresholding, e.g., global thresholding [19], adaptive thresholding with the division of the whole image into smaller parts [20], or optimized thresholding using the Otsu algorithm [21,22,23]. The methods of digital image analysis based on the genetic algorithms [24] and fuzzy logic-based techniques [25] are more effective in a cracks extraction. When there are discontinuities of cracks caused by an image contamination, the Dijkstra algorithm is an effective method of a cracks occurrence prediction [26,27,28]. However, it is a method that requires large computing resources. Very effective in the extraction of the cracking patterns are segmentation methods based on machine-learning algorithms, such as: the Bayesian classifier [29], the AdaBoost classifier [30], or artificial neural networks [31,32,33]. It should be remembered that the full use of the methods based on digital image-processing is rational when they serve some purpose, e.g., an attempt to determine the mechanical properties of a cement composite based on quantitative characteristics of the cracking patterns. There are very few research papers of this type in the literature and this is the main premise for the author of this paper to undertake such a research topic.

The digital image analysis and among them the digital image correlation (DIC) techniques are also used as a research tool in the fracture mechanics of cement composites. Golewski [34,35] conducted research on concrete with the addition of fly ash using the DIC techniques to measure the fracture mechanics parameters, i.e., the stress intensity critical values and the crack tip opening displacement critical values. The results obtained by the DIC method were satisfactory in comparison with the classical measurements. The DIC technique was also used to assess the development of the fracture process zone in concrete [36]. It was found that using image analysis techniques it is possible to trace with high accuracy the development process, length, as well as the crack opening displacement in the fracture process zone. The DIC technique was also used to measure the deformation of compressed concrete [37]. In this way, the non-uniform displacement contour maps were developed, which made it possible to analyze the mutual deformation relations between the concrete structure components. Such analysis would not be possible with the conventional strain measurement. The DIC technique was used also in the study [38] to observe a shear crack in a reinforced concrete beam during a bridge static load test. The measurements allowed for the analysis of displacements depending on the load sequence, in this case truck direction and position as it crosses the bridge. All the aforementioned examples from the literature indicate that using the digital image analysis techniques, the results are very much in line with the measurements made by the conventional methods. In addition, the possibilities of analysis are extended as the use of DIC techniques collects much more computational data on the behavior of the material under given conditions.

Environmental influences, e.g., mechanical, thermal, chemical, etc., make cement composites subject to continuous deformation. Materials based on a cement matrix are characterized by the elastic deformation within a certain load range. Nevertheless, due to their brittleness, as a function of increasing deformations, the strength of the structure is very quickly exceeded locally. This results in the formation of cracks and their further propagation as a function of the time of load exposure and as a function of the increase of this load [39,40,41,42]. Cracks in the cement composite form an extensive system of cracks in its volume and surface, defined as the cracking pattern [11,12,13,14,16,17,43,44,45]. The properties of the structure of the cement composite and the structure of the cement matrix itself make the material very often characterized by lowered but still satisfactory mechanical properties, despite a strongly expanded the cracks system. Unfortunately, with the development of cracks the physical disintegration of the cement composite structure progresses, which results in e.g., decrease of the water tightness, decrease of the frost resistance, decrease of the resistance to elevated temperatures, increase of penetration of aggressive chemical agents into the structure.

In the technology of cement composites it is crucial to know the relations between the cracking patterns and the properties of the cement composite, which, among other things, is the aim of the research described in this paper. This is possible only thanks to a combination of different research techniques, in this case the conventional mechanical testing of the cement matrix in combination with the digital image analysis of the cracking patterns. The tests were carried out on a cement matrix made of a Portland lime cement modified with the addition of microsilica. The material was subjected to a two-phase thermal load at 350 °C and 450 °C. One of the objectives was to develop the original procedure for the localization of cracks on the surface of the cement matrix and to define parameters that could quantitatively characterize the cracking patterns. Thus, an analysis of the cracks structure development process of the modified cement matrix as a function of thermal load was performed. An additional goal was to evaluate relations between the characteristics of the cracking patterns and mechanical properties of the modified cement matrix. This allowed the possibility of estimating the degree of mechanical degradation of the cement matrix structure to be assessed on the basis of measuring quantitative characteristics of the cracking patterns.

## 2. Description of Experiments

### 2.1. Materials and Preparation of Specimens

The subject of the research was a cement matrix made of a Portland lime cement CEM II/A-LL 32.5R. It is a Portland multicomponent cement obtained by joint grinding of the Portland clinker, high-quality specially prepared limestone, and a sulphate additive acting as a regulator of setting time. An important factor in production is the grinding process, which ensures that all components are evenly grounded and homogenized. Thanks to the use limestone, the CEM II/A-LL 32.5R acquires the features characteristic for lime binders, i.e., high and stable quality parameters, moderate hydration heat, a possibility to use it in a wide range of temperatures (including increased resistance to high temperatures), good strength gains in the initial setting period, very good workability of the concrete mix, excellent water bondability in concretes and mortars, good volume stability and lighter colour. The CEM II/A-LL 32.5R has a very wide range of applications, i.e., ready-mixed concrete, the self-compacting concrete, the architectural concrete, prefabrication, for traffic construction (mainly piling and soil stabilization), aerated concrete, and cement and cement-lime mortars. Furthermore, this cement is perfectly suited for the production of concrete mixtures containing the addition of fly ash, as it allows the optimal use of the pozzolanic properties of this additive.

Employing the XRF (energy-dispersive X-ray fluorescence) method, using an Epsilon 3 Panalytical spectrometer, the chemical composition of the CEM II/A-LL 32.5R was determined, which was given as the oxide equivalents: CaO—60.41%, SiO2—18.15%, Al_2_O_3_—5.23%, Fe_2_O_3_—2.70%, MgO—2.49%, SO_3_—2.92%, Cl—0.073%, and Na_2_O—0%. The specific surface area of the cement is 5100 cm^2^/g. In the study there was also used a microsilica (MS) pozzolanic addition, with a specific surface area of about 20 m^2^/g. Two series of cement matrixes were tested:

Classical—CEM II/A-LL 32.5R + water − CP32.5,

Modified—CEM II/A-LL 32.5R + water + 5% MS (by cement weight) − CP32.5 + M.

Within each series, the samples were made in three variants, differing in the *w*/*c* (water/cement) ratio, i.e., *w*/*c* = 0.45, 0.50 and 0.55. A total of 6 different material compositions were tested. The samples of 40 × 40 × 160 mm were tested. They were manufactured in the traditional way, i.e., by mechanical mixing of dry components with water until uniform consistency was obtained. The steel moulds were used to form the samples, in which the cement paste was placed in two layers, consecutively compacted on a vibrating table. The samples were demoulded after 24 h and subjected to a 28-day maturation period, which took place in a climatic chamber (temp. = 20 ± 2 °C, RH = 95–100%). After this period, all the tests described in the paper were carried out.

### 2.2. Thermal-Loading Process

The surface of the samples to be analyzed for the cracking patterns has been pre-aligned with an oscillating grinder. In this way, any surface discoloration that may have occurred during the maturation period was removed. The samples were then subjected to a two-phase thermal load. In the first phase, the samples were placed in a furnace and then heated for 2 h at 350 °C. The pre-heating time of the furnace was about 1.5 h. The cooling time of the samples was about 2 h. After that time, the first scan of cracked surfaces of the cement matrix was performed. Then, in the second phase of the thermal load, the samples were placed again in the furnace and subjected to 2 h of heating at 450 °C. The furnace was pre-heated for a slightly longer time (about 2 h), while the cooling time of the samples until the laboratory temperature was reached (about 20 °C), was about 2.5 h. After the second phase of the thermal load, the same cracked surfaces of the cement matrix were scanned again and the obtained images were quantitatively analyzed using an image-processing method. The samples were then subjected to the destructive tests to determine the basic mechanical characteristics of the cement matrix.

**Figure 1:**
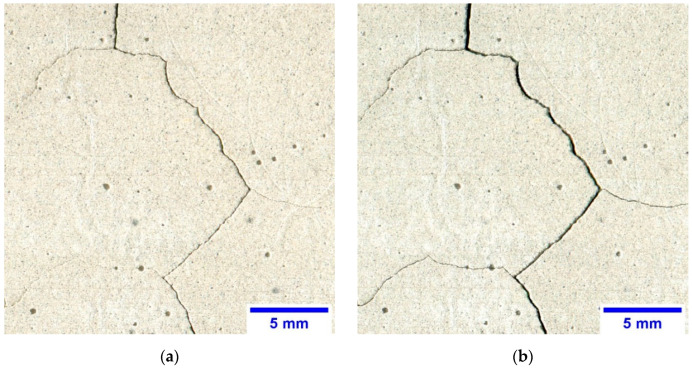

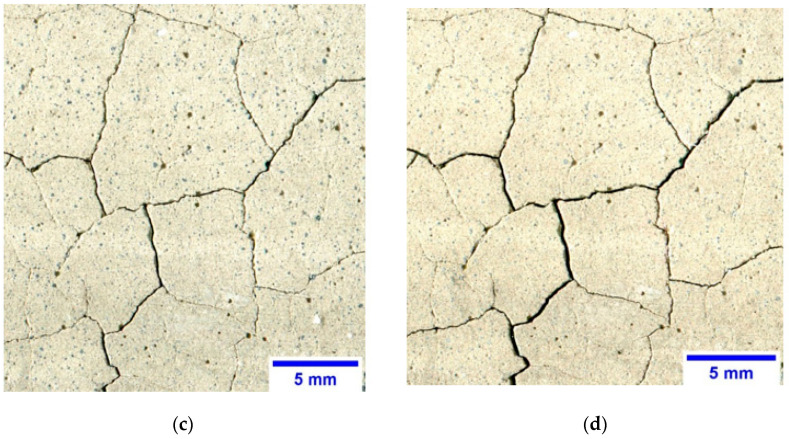
The cracking pattern visible on the surface of the cement matrix with *w/c* = 0.55: (**a**) CP32.5—the first phase of thermal load; (**b**) CP32.5—the second phase of thermal load; (**c**) CP32.5 + M—the first phase of thermal load; (**d**) CP32.5 + M—the second phase of thermal load.

Cracks visible on the surface of the cement matrix after the thermal loading are the result of several factors, i.e., deformations in the form of an increase in material volume due to the heating, shrinkage due to the cooling, bursting action of water vapor under pressure, and chemical transformations in the form of decomposition and dehydration of some cement phases. However, in the range of thermal interaction from room temperature to 450 °C, the most cracks that form and become destructive, are caused by the physical changes of the cement matrix occurring in the form of deformations [46,47,48]. Figure 1 shows the same fragments of cracked surface of the cement matrix. There is a clear difference in the morphology of the cracking pattern between the two series as well as between the first and second phase of the thermal load.

### 2.3. Determination of Basic Mechanical Properties

As part of the tests, the tensile strength (*f_cf_*) was tested in a three-point bending scheme according to the EN 12390-5 [49]. The test was carried out in two variants, on the reference specimens (after 28 days of maturation)—*f_cf(R)_*, and on specimens subjected to the thermal load—*f_cf(T)_*. The results presented in the further part of the study are the arithmetic mean of 3 samples. The compressive strength (*f_c_*) was tested on halves of the samples obtained after the *f_cf_* testing, according to the EN 12390-3 [50]. The test was also carried out in two variants, i.e., for the reference samples (*f_c(R)_*) and thermally loaded (*f_c(T)_*) ones. In the case of *f_c_*, the results are the average of the 6 samples. The *f_cf_/f_c_* ratio, which according to [51] is the basic measure of brittleness of a cement composite, was also analyzed.

### 2.4. Cracking Patterns Detection and Analysis

The cracking patterns formed after the thermal load were the subject of major research. The whole analysis process was carried out on the digital images of the cracked surface of the cement matrix. The cracking patterns were examined both after the first and second phase of the thermal load. The surface of the samples was scanned on an optical scanner. The same surface was always analyzed. Taking into account the direction of the samples formation it was the bottom surface because it was the most even and free of discoloration. The cracked surface itself was scanned in a high 1200 DPI optical resolution, and in the RGB colour mode. The lossless TIF format was used for recording. Scanning in the above resolution is equivalent to covering the surface with 47.2441 pixels/mm, which gives great opportunities to identify cracks with very small opening widths. The whole process of digital image analysis has been performed in the open-source ImageJ software. The software is characterized by very high possibilities of analysis and is very popular in many scientific fields [52,53,54,55].

#### 2.4.1. Image Double-Segmentation Method

In order to be able to analyze the cracking patterns, a procedure of double-segmentation of the image has been developed, the aim of which is digital extraction of cracks. The procedure was developed using the Trainable Weka Segmentation (TWS) tool [56], which is a part of a large extension of the ImageJ—the Fiji. The TWS is a segmentation module using machine-learning algorithms. The aim of the developed procedure was to produce the pixel-based segmentations, which will unambiguously (binary) show the cracking pattern against the remaining surface of the cement matrix. The developed procedure consists of the following steps:

- verification of the original image position—during the scanning process, the position of the longitudinal axis of the sample was attempted parallel to the scanning crosshead; in case of deviation from this assumption, the rotation correction was performed digitally;
- definition of the fixed analysis area—individual samples may have differed slightly in dimensions as the cement matrix was subjected to thermal deformations, and in addition there may have been slight deviations between the dimensions of the moulds; for quantitative analysis of the cracking patterns to be correct a fixed analysis area had to be adopted, which was defined as 157 mm × 38.5 mm rectangle; each individual image was cropped to this dimension;
- creation of a digital grayscale image—at this stage, the image was converted from the original red, green and blue (RGB) mode to the 8-bit grayscale;
- implementation of the sharpen filter—in order to sharpen the image for analysis, the sharpen filter built into the ImageJ was used, which slightly increases contrast and accentuates details on the image; the disadvantage of the filter is also a slight accentuation of possible noise; the filter works by replacing each single pixel with a 3 × 3 pixel weighted average according to the following weight:

$$\left[\begin{array}{ccc} -1 & -1 & -1\\ -1 & 12 & -1\\ -1 & -1 & -1 \end{array}\right]$$

- the first stage of the image segmentation—at this stage, using TWS, a first stage segmentation pattern was created; the purpose of segmentation was to classify all pixels forming the image into two defined classes, i.e., cracks—class 1, background—class 2; the following training features were used: the difference of Gaussians, Sobel filter, membrane projections, hessian, and Gaussian blur; the membrane thickness was set to 1, the membrane patch size—19, minimum sigma—1.0, maximum sigma—16.0; for teaching the classifier of the first stage two random surface fragments (one each of CP32.5 and CP32.5 + M series) with dimensions of 1000 px × 1000 px each were used;
- creating a binary image after the first stage of segmentation, where the cracks were classified as black (0 on the histogram) and the remaining area as white background (255 on the histogram);
- creating a base image for the second stage of segmentation—for this purpose, two different characters of the same image were added together, i.e., the first character—the image resulting from the first stage of segmentation; the second character—the primary image on an 8-bit greyscale, contrasted in such a way that the maximum of the range of the new histogram was in the value of the gradient for which the primary histogram reaches its maximum counts, while the minimum value of the histogram range remains unchanged;
- the second stage of the image segmentation—carried out on a previously made base image; the classifier of the second stage of segmentation was developed on the same surface fragments as the classifier of the first stage; the same settings of training features as during the first stage were used to teach the classifier;
- creating a final binary image showing the cracking patterns (black pixels) separated from the rest of the sample (white pixels).

Each single surface has been treated according to the image double-segmentation procedure presented, using the classifiers created for the first and second stage, respectively. Then, a quantitative analysis of the cracking patterns was carried out on the images prepared in this way.

**Figure 2:**
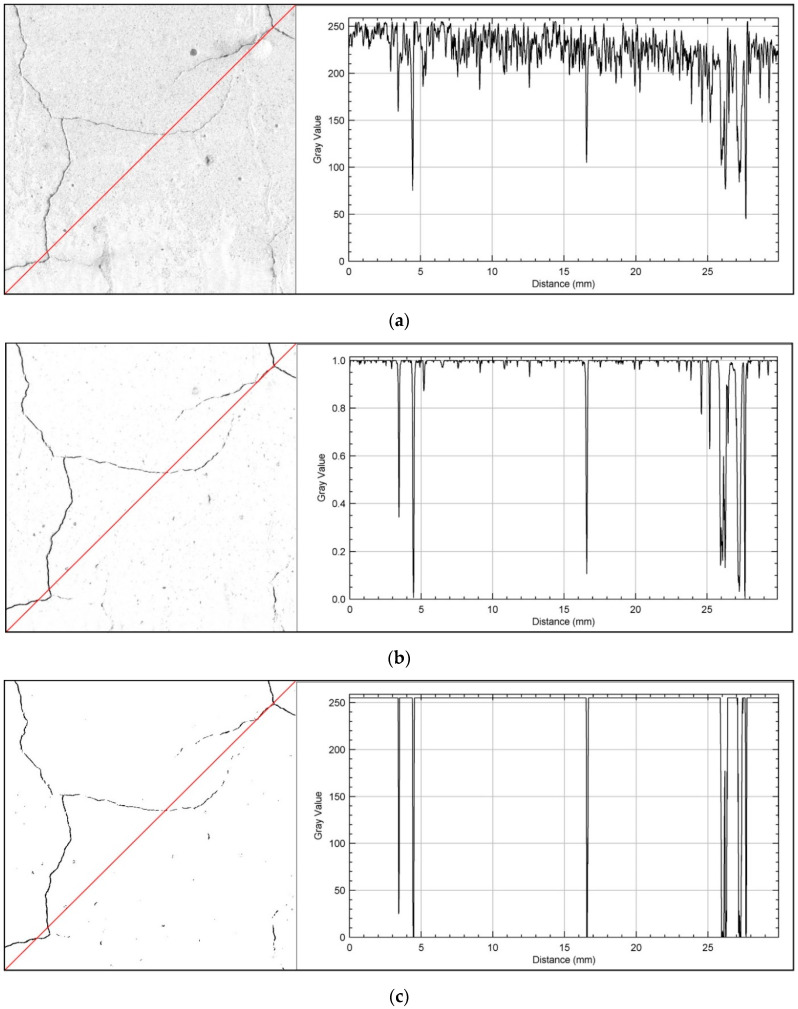
The surface of the pattern for CP32.5 series together with a plot profile showing the gradient value on the greyscale along the red line—the first segmentation stage: (**a**) input image; (**b**) probability map; (**c**) final image.

Figure 2 shows a fragment of the pattern image for the CP32.5 series during the first stage of segmentation, together with a plot profile along the red line in the image. Analyzing the plot profile, the input image shows a large noise gradient associated with the background, oscillating mainly between values of about 180–255 in the histogram. On the plot profile on the probability map, the amount of noise has been significantly reduced, and the visible peaks in the graph represent the place where the cracks occur along the length of the plot line. On the final image after the first stage of segmentation the cracks forming the cracking pattern are well extracted, however, there are numerous visible impurities in the image resulting from discolorations on the sample surface.

**Figure 3:**
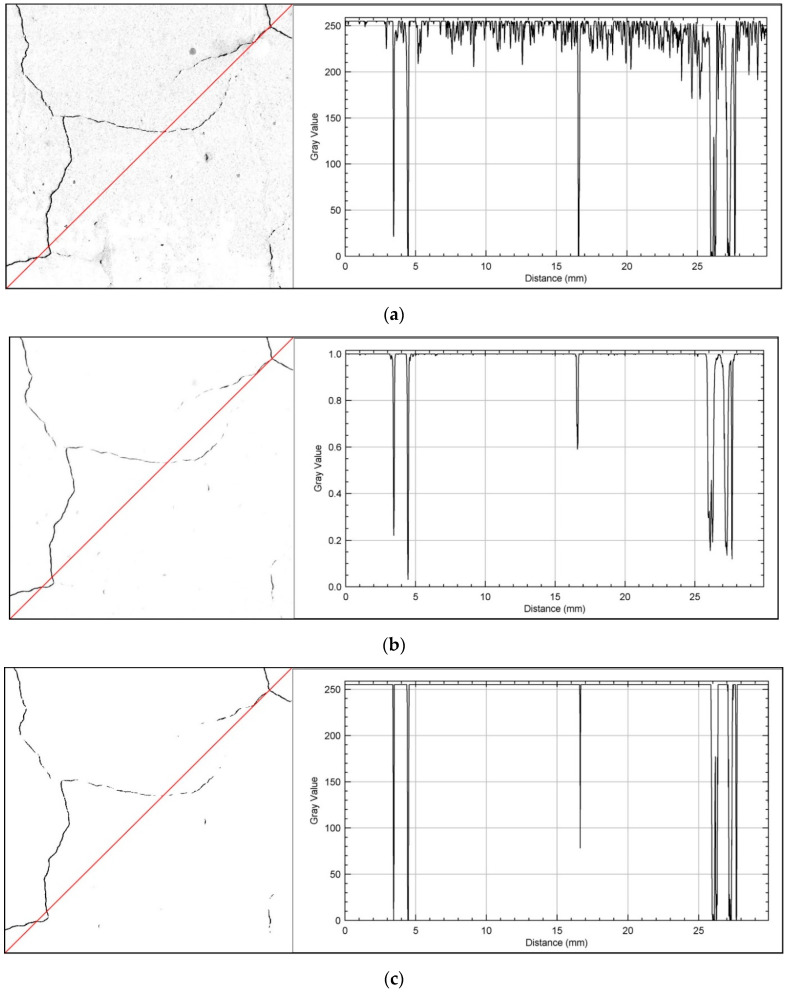
The surface of the pattern for CP32.5 series together with a plot profile showing the gradient value on the greyscale along the red line—the second segmentation stage: (**a**) input image; (**b**) probability map; (**c**) final image.

Figure 3 shows the course of the second stage of segmentation for the same pattern area. The plot profile of the input image shows a much clearer contrast between the existing cracks and the background. The noise range of the background (from about 210–255 on the histogram) is much lower compared to the first stage of image segmentation, which translates into much more accurate extraction of cracks from the surface. On the probability maps, the value of background noise has been reduced to practically zero. The final image after the second stage of segmentation is characterized by binary separation of the cracking patterns from the remaining surface of the sample, with a much lower level of image contamination compared to the first stage of segmentation.

#### 2.4.2. Determination of Cracking Patterns Parameters

In order to quantify the cracking patterns three parameters were defined:

- total crack area—TCA;
- crack density—*CD*;
- fractal dimension of cracking patterns—*CP-D_B_*.

The results of the parameters describing the cracking patterns presented below are the average of the measurements made on 3 samples for each series and the *w/c* ratio. The total crack area is defined as the area occupied by cracks up to the total area of the analyzed area. It is a parameter that allows to directly determine the degree of surface cracking. It is calculated according to the formula:

(1) $$TCA=\frac{A_{C}}{A_{I}}\cdot 10000\left[\frac{{\mathrm{cm}}^{2}}{m^{2}}\right]$$

where:

- $A_{C}$—area occupied by cracks [px], i.e., number of black pixels (0 on the histogram);
- $A_{I}$—total area of the analyzed area [px]; in each case, the analyzed area had dimensions of 157 mm × 38.5 mm, i.e., 7418 px × 1819 px (the scanning resolution—1200 DPI); $A_{I}$ = 13,493,342 px;
- 10000—a multiplier to show the result in $\left[\frac{{\mathrm{cm}}^{2}}{m^{2}}\right]$.

A measure of the concentration of cracks on the material surface is *CD*. The parameter is defined as the number of single cracks appearing on the length of the analyzed surface [8,12]. On the surface of each sample three test lines were determined, which divided the sample surface into 4 equal parts in the longitudinal direction. The *CD* measurement was carried out along the test lines, thus the result for a single sample is the average of three determinations. The *CD* is defined according to the formula:

(2) $$CD=\frac{\frac{\sum_{i=1}^{n}N_{C,i}}{n}}{L_{S}}=\frac{\bar{N_{C}}}{L_{S}}\;\;\left[\frac{1}{m}\right]=\left[m^{-1}\right]=\left[\frac{\mathrm{cracks}}{m}\right]$$

where:

- $L_{S}$—length of the test line; length of the analyzed surface in the longitudinal direction;
- $L_{S}=0.157\;m$;
- $N_{C,i}$—number of cracks intersecting the *i*-th test line;
- n—number of test lines on a single sample; n=3.

The parameter that describes the degree of complexity of the difficult to describe flat and spatial structures, in terms of morphology, is the fractal dimension. Recent scientific research shows a growing interest in the fractal approach to analyzing the cracking patterns [57,58,59]. This approach requires the assumption that the cracks system on the surface of the material is a fractal, i.e., it has some fractal characteristics—it is shapeless (unambiguous shape is impossible to determine), an inability to describe the form with a mathematical relationship (only recursive dependency), and self-similarity (the isolated fragment is similar to a larger whole) [60,61]. Observing the cracking patterns on the surface of cement composites, it can be stated that all the above assumptions are met.

**Figure 4:**
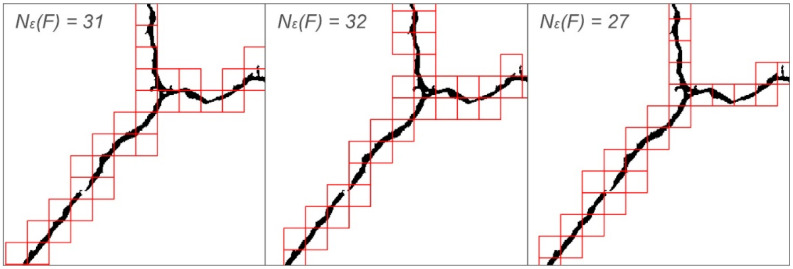
Number of boxes containing significant pixels at different grid orientation positions, for the same calibre of the grid.

In the study, the fractal dimension of the cracking patterns (*CP-D_B_*) by the box-counting method was measured. The FracLac plugin for ImageJ was used [62]. For a single sample, the measurement was performed 12 times, after which the final result was the average of these measurements. Each single measurement was taken at a different grid orientation. This results in a more reliable result for the whole sample. It may happen that with the same calibre of the grid, the number of boxes containing significant pixels will be different depending on the grid orientation, as shown in Figure 4. During the measurements, a linear increment of the grid calibres was used, when sampling in 100 different grid sizes, i.e., from 5 to 818 px. The size of the largest sampling element was defined as 45% of the length of the shorter edge of the image. Graphically, the fractal dimension is defined as the slope of the regression line on a graph that shows the number of boxes containing significant pixels at different grid sizes as a function of the grid size. This is equivalent to the equation:

(3) $$D_{B}=\underset{\varepsilon \to 0}{\lim}\frac{\log N_{\varepsilon }\left(F\right)}{\log\left(1/\varepsilon \right)}\;\;\left[-\right]$$

where:

- $N_{\varepsilon }\left(F\right)$—number of boxes containing significant pixels at different ε grid sizes,
- ε—size of the grid.

## 3. Experimental Results

### 3.1. Mechanical Properties of Cement Matrix—f_c_, f_cf_, f_cf_/f_c_

Table 1 presents average values of the mechanical parameters of lime cement matrix, i.e., *f_c_*, *f_cf_*, and *f_cf_/f_c_*. The properties were determined for the both reference samples and thermally loaded samples. Decrease of the values of mechanical parameters, in the form of percentage differences due to the thermal loading of the cement matrix, was also shown. Table 2, on the other hand, presents the values of coefficients of variation of individual parameters, which allows the quality and repeatability of the tests to be assessed. In case of *f_c_*, the values of the coefficient of variation in each case were lower than 10%, which is a small and acceptable scatter of results. However, for *f_cf_* in two cases, the value slightly exceeded 10%, but this does not indicate a badly conducted research process. The quality and repeatability of the obtained results of mechanical properties is at a very good level.

### 3.2. Cracking Patterns Characteristics—Total Crack Area (TCA), Crack Density (CD), and Fractal Dimension of Cracking Patterns (CP-D_B_)

**Figure 5:**
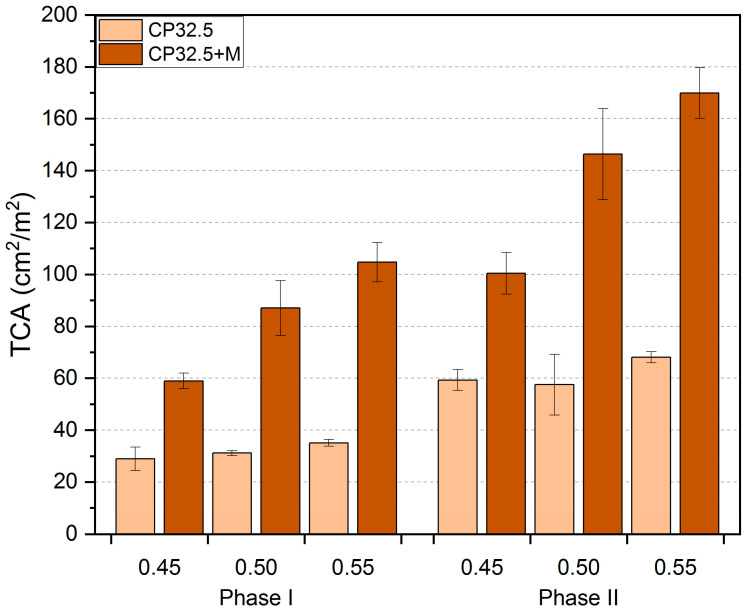
Results of the total crack area (*TCA*) of the lime cement matrix after the first and second phase of thermal load; error bars show standard deviation.

**Figure 6:**
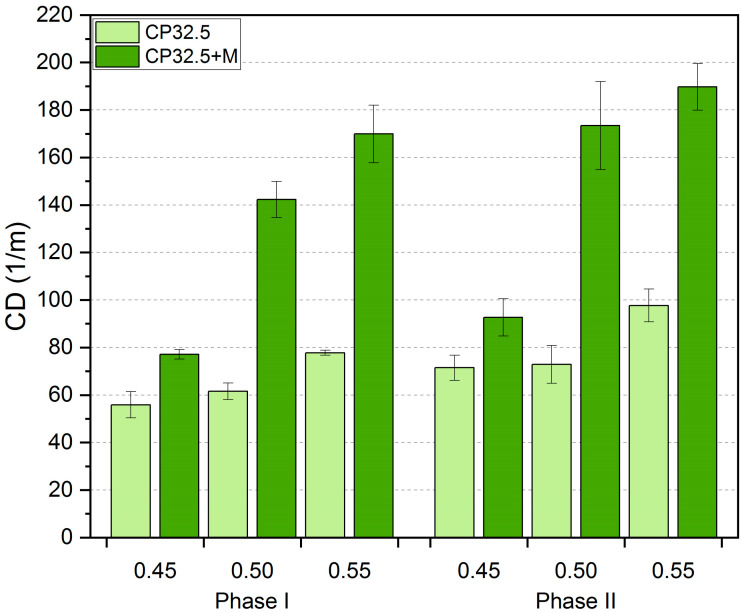
Results of the crack density (*CD*) of the lime cement matrix after the first and second phase of thermal load; error bars show standard deviation.

**Figure 7:**
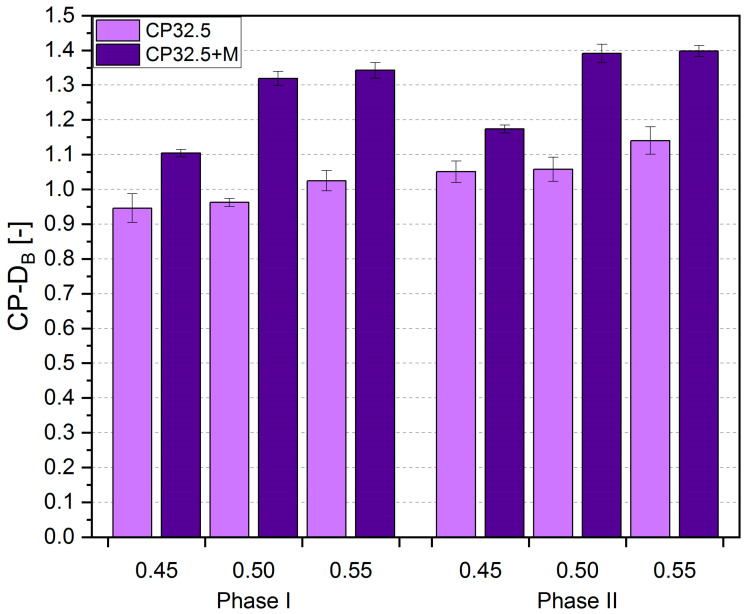
Results of the fractal dimension of cracking patterns (*CP-D_B_*) of the lime cement matrix after the first and second phase of thermal load; error bars show standard deviation.

The *TCA*, *CD* and *CP-D_B_* values obtained from the image analysis, together with the standard deviation, are shown in Figure 5, Figure 6 and Figure 7, respectively. For the purpose of the analysis, the results obtained for particular series have been presented in a division into the thermal load phase and the value of the *w/c* ratio. The values of standard deviations indicate small variability of the results and thus their good quality and accuracy.

## 4. Discussion

### 4.1. Mechanical Properties of Thermally Loaded Cement Matrix

The technological parameter that mainly determines the properties of the hardened cement matrix is the *w/c* ratio. As it increases, the share of cement in the material volume decreases and the amount of water increases. Immediately after mixing the binder with water, the cement paste is a liquid mixture that can be considered a dispersion system [63]. In this system, initially the cement grains are suspended in an aquatic environment. During the binding and hydration process, the hydrate crystals grow slowly on the surface of cement grains, which at some point start to grow together and bind to form a rigid structure capable of carrying loads as a function of time [64,65,66]. As the amount of water in the material volume increases at the expense of cement, the distances between cement grains increase. Thus, the strength gain of the cement matrix is slower because more time is needed for the hydrates on the adjacent cement grains to start to grow together during the hydration process. In addition, the number of interconnections between the hydrates is much smaller than in a cement paste system with a low *w/c* ratio. During the progressing of hydration, a certain part of water evaporates from the space, leaving empty spaces which are defects of the cement matrix. Taking into account fewer connections between the hydrates and more voids in the high *w/c* cement matrix, the strength of the cement skeleton is reduced.

This was reflected in the research carried out. The cement matrix obtained lower *f_c_* and *f_cf_* values along with an increase in the *w/c* ratio in all cases, i.e., for the reference samples and those subjected to the thermal load. In the case of reference samples with *w/c* = 0.50 and 0.55, the *f_c(R)_* values were 15.4% and 28.3% lower, respectively, than the samples with *w/c* = 0.45. For *f_c(T)_* the above dependence assumed values equal to 14.3% and 42.0%, respectively. In terms of the tensile strength of the cement matrix, the samples with *w/c* = 0.50 and 0.55 had lower *f_cf(R)_* values by 14.1% and 31.8%, respectively, than the samples with *w/c* = 0.45. After thermal loading, the differences in *f_cf(T)_* were higher and amounted to 27.2% and 37.1%, respectively. While in the case of the reference samples the decrease of *f_c(R)_* and *f_cf(R)_* together with the increase of the *w/c* ratio is comparable, the thermal load causes bigger relative decreases of *f_cf(T)_* than *f_c(T)_*, especially for a cement matrix with a more concentrated dispersion system, i.e., with lower *w/c* ratio. This state results directly from the brittleness of the material and the increase in this property due to the thermal loading.

Bearing in mind the nature of the thermal load, i.e., a two-phase process at two different temperatures (350 °C and 450 °C), the deterioration in the mechanical properties of the lime cement matrix was not as great as it could be in the case of traditional Portland cement. According to EN 197-1 [67], the Portland multicomponent cement, CEM II/A-LL, is produced by burning 80–94% of the Portland clinker together with 6–20% by weight of specially prepared limestone. The limestone itself has a very good thermal resistance, so its addition to the cement will also improve this property for the cement matrix. The drop of *f_c_* after thermal load was in the range of 34.0–53.9% depending on the *w/c* ratio and the presence of MS. These drops were slightly lower for the MS modified cement matrix (CP32.5 + M). In the case of *f_cf_*, the deterioration of this parameter after thermal loading was very similar in each case, thermal deterioration of *f_cf_* ranged between 70.2–79.6%. The reduction of cement matrix cohesion in the course of the thermal load is a result of chemical and physical transformation of its structure. Chemically, the cement matrix is very stable at the temperatures applied in the tests. The main component of the hardened cement matrix, which at the same time is most responsible for the chemical cohesion of the material, i.e., CSH phase, disintegrates from the temperature of about 600 °C [47,48]. In the analyzed temperature range, the main destructive factor is the physical transformation of the cement matrix, i.e., the thermal deformation and water vapour pressure gradient in the material. Both of these phenomena are connected with the occurrence of high values of local stresses, which leads to exceeding the local tensile strength of the cement matrix and thus to the formation of cracks. The discontinuities of the structure in the form of cracks are the main and direct cause of deterioration of mechanical properties of the cement matrix subjected to a thermal load.

Modification of the cement matrix with MS resulted in an increase in *f_c_* for the both reference samples and those subjected to the thermal load. The mechanism of action of MS consists in reacting with Ca(OH)_2_, which results in the formation of a secondary CSH phase, which additionally seals the structure of the cement matrix and increases the degree of mutual binding of cement hydration products with each other. This reaction is the quicker and more intense the finer the MS grains are. The CP32.5 + M series was characterized by higher *f_c(R)_* and *f_c(T)_* values by 23.4% and 41.8%, respectively, than CP32.5 series. The differences were greater the higher the *w/c* ratio was. In the case of *f_cf_*, the effect was the opposite as the addition of MS intensifies the process of brittle cracking of the cement matrix [13]. Thus, the MS modified samples, i.e., CP32.5 + M obtained lower values of *f_cf(R)_* and *f_cf(T)_* than classical cement matrix (CP32.5), by 27.1% and 36.2%, respectively.

The *f_cf_/f_c_* ratio is a very simple measure of material brittleness. According to [51], a material which *f_cf_/f_c_* ratio is less than 0.125 is considered as brittle. According to this criterion, all tested variants of the cement matrix are considered as brittle. It was not noticed that the *w/c* ratio unambiguously determines this parameter because among the reference samples of the CP32.5 series the brittleness of the material decreases with the increase in *w/c*, whereas for the CP32.5 + M series the opposite relation occurs. Within each series, these trends do not occur for samples subjected to the thermal load. Modification of the lime cement matrix with MS resulted in an increase in the brittleness of the structure by 40.4% and 54.1%, respectively, for reference samples and those loaded with the elevated temperature, as compared to the CP32.5. However, the effect of thermal influence on *f_cf_/f_c_* varied from series to series, and so for the CP32.5 the average increase in brittleness was in the range of 47.0–58.8%, while for the CP32.5 + M series it was 56.2–70.7%. The obtained brittleness results confirm the above conclusions concerning *f_c_* and *f_cf_*.

### 4.2. Morphological Characterization of Cracking Patterns

The total area occupied by cracks is expressed by the *TCA* values (Figure 5). The factor that had the greatest influence on this parameter was the MS addition. The use of this pozzolanic additive reduces the resistance of the cement matrix to brittle cracking, thus less energy has to be supplied to the system to initiate the cracking process. The thermal load caused significant stresses in the structure of the cement matrix related to its thermal deformability. Thus, the MS-modified cement matrix (CP32.5 + M) had higher *TCA* values on average by 160.3% than CP32.5, for the first phase of the thermal load. After the second phase of the thermal load the difference between the series was 124.4%. Thus, the addition of MS increased the area occupied by cracks more than twice. It would seem that the cement matrix with increased mechanical strength (CP32.5 + M) should crack less. However, in terms of the mechanical strength of the whole system, such parameters as scattering of cracks on the surface of the material and an opening width of the cracks should also be considered.

The influence of the degree of cement grain concentration (*w/c*) in the cement matrix system was more noticeable in the case of samples modified with MS (CP32.5 + M). The global trend indicates an increase in *TCA* as the *w/c* increases. In the case of the first phase of thermal load, for the CP32.5 series samples with *w/c* = 0.50 and 0.55, they had higher *TCA* values on average by 7.7% and 21.2% than samples with *w/c* = 0.45. The same dependence but for the CP32.5 + M series was equal to 47.7% and 77.7%, respectively. In the case of the second load phase, the CP32.5 series samples with *w/c* = 0.50 obtained slightly lower TCA values (by 3%) compared to the samples with *w/c* = 0.45. It should be noted, however, that in this case relatively high standard deviation values were also obtained. On the other hand, the global trend was maintained for the samples with *w/c* = 0.55, which had higher *TCA* values on average by 14.9% than samples with *w/c* = 0.45. For the CP32.5 + M series, *TCA* values for the samples with *w/c* = 0.50 and 0.55 were on average 45.8% and 69.2% higher from samples with *w/c* = 0.45. The increase in *TCA* along with the increase in *w/c* can be associated with a higher amount of water closed in the structure of the cement matrix as well as a higher porosity of the cement paste due to the evaporation of the excess water during the maturation period. During the thermal load the deformation of the cement matrix is greater the more water that is closed in the structure of the material. An increase in the possibility of deformation of the structure results in the formation of the cracking pattern, characterized by a larger surface area occupied by cracks.

The increase in the deformation of the cement matrix subjected to the repeated thermal load resulted in further development of the already existing cracks. After the second phase of the thermal load, the *TCA* values for the classical cement matrix (CP32.5) was about twice as high as in the first phase (Table 3). Modification of the cement matrix with MS had a positive effect on limiting the relative degree of development of surface crack structure between the first and second phase of the thermal load. The relative *TCA* increases were lower than those of the CP32.5 series and ranged from 62.2–70.4%.

The degree of crack dispersion on the cement matrix surface is represented by the *CD* values (Figure 6). A higher *CD* value indicates the occurrence of more cracks on a unit length of the material. Analyzing the results obtained, a clear upward trend of *CD* was observed along with an increase in the *w/c* ratio. Samples of the CP32.5 series with *w/c* = 0.50 and 0.55 obtained on average higher *CD* values by 6.1% and 37.9%, respectively, from samples with *w/c* = 0.45. The above differences for the CP32.5 + M series are much greater and have the following values—85.7% and 112.4%. Such results can be related to the fact that a cement matrix with a higher degree of cement grain concentration is characterized by higher cohesion. In such a system, the number of connections between the hydrates is much higher and the intermolecular interaction forces are stronger due to smaller distances between the hydrated cement grains. Much more energy needs to be supplied to such a compacted system in order to achieve a stress state that allows the process of brittle cracking and crack propagation to begin.

The addition of MS to the lime cement matrix caused a significant increase in the *CD* value. In the case of the first phase of the thermal load, the CP32.5 + M series samples achieved higher *CD* values than the CP32.5 series on average by 95.7%. After the second phase of the thermal load the difference between the series was 87.3%. Thus, the difference in the number of cracks per unit length of material was twofold. The results of the mechanical properties discussed above indicated a significant increase in the brittleness of the CP32.5 + M series in relation to the CP32.5, and most likely this property is responsible for a much higher number of surface cracks. The process of cracking in the case of cement matrix with higher brittleness (CP32.5 + M) is initiated with a lower value of a load that affects the material. Therefore, under the same thermal load conditions, the more brittle cement matrix will be characterized by more cracks.

Between the first and second phase of the thermal load, the existing cracks not only continued to propagate and develop, but new cracks also appeared on the surface of the cement matrix. As with *TCA*, the relative increase in *CD* values (Table 3) was lower for the CP32.5 + M (11.7–21.9%) compared to the CP32.5 (18.4–27.8%). It was not observed that the size of increments depends on the degree of concentration of cement grains in the cement matrix.

**Figure 8:**
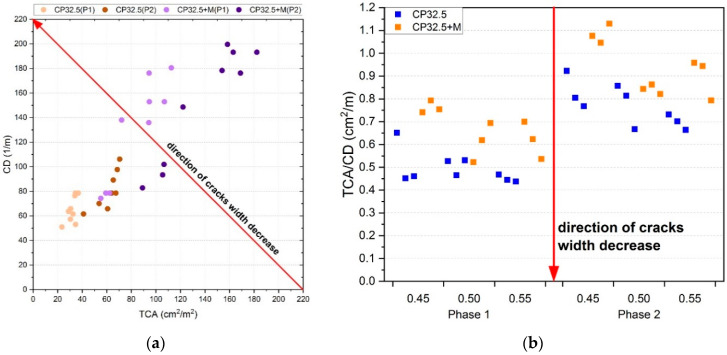
The relative opening width of the crack described as: (**a**) *CD(TCA)* function; (**b**) *TCA/CD* ratio; for series designation: (P1)—after the first phase of the thermal load; (P2)—after the second phase of the thermal load.

The parameter that determines to a large extent the degree of degradation of the cement composite is the width of the crack opening. From the data obtained from the image analysis, this property can be relatively presented in the form of the relation between *TCA* and *CD*, as shown in Figure 8a. When the surface occupied by cracks decreases with a simultaneous increase in the density of cracks, this indicates that a single crack has an increasingly smaller surface area, i.e., the width of its opening relatively decreases. The direction of change of this relationship is shown on the figure as a red arrow. The relative average opening width of the crack can also be shown as the *TCA*/*CD* ratio (Figure 8b), which is a more transparent representation of the change of this property and gives the possibility to make a comparative analysis. The results of the *CD* indicate that with the repeated thermal load, new cracks are created in a certain number, but mainly develop and propagate the existing ones. A situation of this type is consistent with the principles of the fracture mechanics, because much more energy has to be supplied to the system in order to create a new crack, compared to the amount of energy, which causes the propagation of the already existing crack [41,68,69]. The analysis performed indicates that after the second phase of the thermal load the relative average crack opening width is higher by as much as 47.8% in comparison with the state after the first phase.

The cement matrix modified with MS (CP32.5 + M) achieved on average 34.9% and 22.3% higher relative crack opening widths than the classical cement matrix (CP32.5), respectively after the first and second phase of the thermal load. This state can be linked to the higher brittleness of the CP32.5 + M series. Despite higher relative crack widths, the CP32.5 + M series samples achieved higher *f_c_* values because when the cement matrix is compressed the effect of overlapping of individual fragments of the cracked material may appear, which does not affect this mechanical property. On the other hand, in the case of stretching the cement matrix, the presence of cracks with larger opening widths has a negative effect. In the case of the *f_cf_* testing in the three-point bending scheme, the failure zone runs through the cross-section which is characterized by the least local mechanical cohesion.

The fractal dimension is a quantity that expresses the degree of development and complexity of the analyzed structure [61]. *CP-D_B_* for the cement matrix tested is shown in Figure 7. Analyzing the results, an upward trend of *CP-D_B_* was observed along with an increase in the *w/c* ratio. Thus, the samples with the *w/c* = 0.50 and 0.55 had on average higher *CP-D_B_* values by 1.2% and 8.4%, respectively, than the samples with *w/c* = 0.45, in case of the CP32.5 series. The differences for the CP32.5 + M series were 19.0% and 20.3%, respectively. A higher value of the fractal dimension is equivalent to a higher degree of complexity of the cracking pattern. The MS addition had a very significant effect on *CP-D_B_* as the CP32.5 + M series samples had higher values of this parameter than the CP32.5 series on average by 28.3% and 21.9%, respectively, after the first and second phase of the thermal load. As before, this effect can be associated with an increase in the brittleness of the MS cement matrix, which promotes the propagation of cracks under the thermal load conditions.

Analyzing the development of the cracking pattern between the first and second phase of the thermal load, the same relationship was noted as for *TCA* and *CD* (Table 3). Despite a much more developed cracks structure in the CP32.5 + M series, the addition of MS relatively limited the development of cracks after repeated thermal loading. The increase in *CP-D_B_* values for the CP32.5 ranged from 9.9–11.2%, while for the CP32.5 + M the increase was half that and was equal to 4.1–6.3%. No clear influence of the degree of concentration of cement grains on relative differences in *CP-D_B_* between the first and second phase of the thermal load was observed. The use of *CP-D_B_* to describe the surface structure of cracks is very beneficial from the statistical point of view. Out of all three parameters, i.e., *TCA*, *CD*, *CP-D_B_*, the results of *CP-D_B_* are characterized by the lowest variability because the coefficients of variation for *TCA* ranged from 3.1–20.3%, for *CD* 1.3–11.0%, and for *CP-D_B_* 0.9–4.4%. This means that even small but statistically significant differences in the structure of the cracking patterns between samples can be captured with very high accuracy.

**Figure 9:**
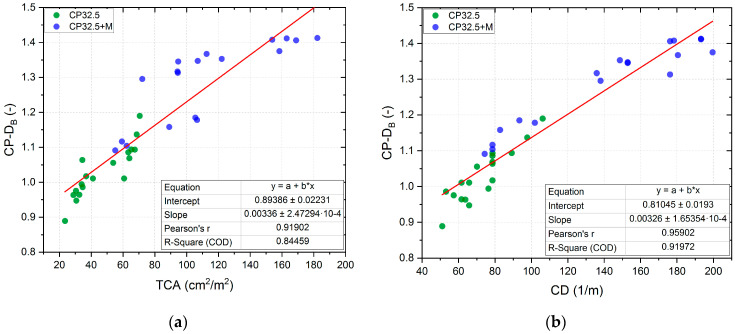
Analysis of the linear regression of *CP-D_B_* in function of: (**a**) *TCA*; (**b**) *CD*.

*CP-D_B_* is very strongly correlated with the other two parameters describing the cracking patterns, i.e., *TCA* and *CD*. Globally, the increase in *CP-D_B_* is accompanied by an increase in the other two parameters. The Pearson correlation coefficient between the *CP-D_B_* and *TCA* is 0.92, and between *CP-D_B_* and *CD* is as much as 0.96. Both of these coefficients are statistically significant at a level of statistical significance α = 0.05. Thus, the change in the degree of development of the cracking patterns on the surface of the lime cement matrix is reflected in almost the same change in *TCA* and *CD*. The relationships between these parameters (Figure 9) allow for a very accurate estimation of *CP-D_B_*, based on the knowledge of one of the other two parameters describing the cracking patterns. Using the least squares method (LSM), linear regression equations with diagnostic statistics were calculated. The obtained regression lines cover empirical data very well, because for *CP-D_B_(TCA)* the fit of regression line is 84.5% and for *CP-D_B_(CD)* 92.0%. Such a high degree of alignment of linear regression lines is of great practical importance, because for estimation purposes it is possible to measure only one of the parameters of the cracking patterns of the cement matrix in order to determine the other two parameters with very high accuracy. This approach reduces the time needed for the image analysis.

### 4.3. Relationship between Cracking Patterns Properties and Mechanical Features—Estimation Possibilities

In order to assess the strength of the correlations between the parameters describing the cracking patterns and the mechanical properties of the lime cement matrix, Pearson (*r*) and Spearman (*ρ*) correlation coefficients were calculated, the values of which are summarized in Table 4. The application of these two different correlation coefficients results from the fact that the classic Pearson correlation coefficient measures the linear relationship between the variables, while the Spearman correlation coefficient indicates any monotonous relationship, which does not have to be linear. The interpretation of both these correlation coefficients is the same, while comparing their values allows to assess whether the analyzed relation is more linear or non-linear. Additionally, the Spearman correlation is not sensitive to outliers. The analysis of the results obtained showed that the correlations between compressive strength (*f_c_*) and parameters of the cracking patterns are generally weak or low, and they are not statistically significant. The *f_c_* estimation from these parameters would be pointless due to the generation of very large errors.

The relationship between brittleness (*f_cf_/f_c_*) and the parameters of the cracking patterns has a moderate, high, or very high correlation, while a very high correlation was achieved between *f_cf_* and the parameters of the cracking patterns. Tensile strength estimation based on the measurement of the cracking patterns characteristics would be quite accurate. Both *f_cf_/f_c_* and *f_cf_* are negatively correlated with the parameters of the cracking patterns, i.e., as the mechanical properties of the lime cement matrix increase, the surface area occupied by the cracks, their number and complexity will be reduced. The strongest correlations for the Pearson correlation were obtained for *CD_(P1)_-f_cf(R_*_)_, *CP-D_B(P1)_-f_cf(T)_*, and *TCA_(P1)_-f_cf(R)_/f_c(R)_*—for these three relations |r|=0.87. In the case of the Spearman correlation, the strongest relations are *CD_(P1)_-f_cf(R)_*, *CP-D_B(P1)_-f_cf(T)_*, and *CP-D_B(P2)_-f_cf(T)_*—for the first two dependencies |ρ|=0.88 and for the third one |ρ|=0.89.

**Figure 10:**
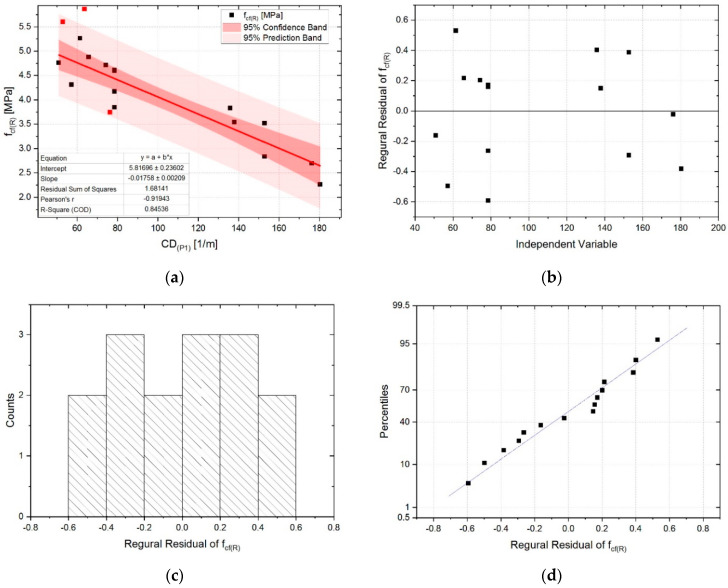
Results of the linear regression analysis for the *CD_(P1)_-f_cf(R)_* relationship together with residual plots: (**a**) regression line (red points—outliers); (**b**) residuals vs. independent; (**c**) histogram of residuals; (**d**) normal probability of residuals.

For the two most strongly correlated relationships, i.e., *CD_(P1)_-f_cf(R)_* and *CP-D_B(P1)_-f_cf(T)_*, a linear regression analysis was carried out and the evaluation was performed of the degree of accuracy of tensile strength estimation for both standard samples and those degraded by the influence of elevated temperature. Figure 10 shows the results of regression analysis together with residual plots for the *CD_(P1)_-f_cf(R)_* relation. During the analysis 3 outliers were identified, which differed significantly from the fitted regression line—they were marked in red and were not included in the analysis. The exclusion of these points from the analysis resulted in an increase in the Pearson correlation coefficient, in the negative correlation range, from −0.87 to −0.92. Although two of the three outliers points were within the 95% prediction band, they were characterized by the highest residuals values. The final linear regression line obtained is characterized by a very good degree of fitting to the empirical data, which are covered in 85% (R-Square = 0.85). Equation coefficients, both slope and intercept, are statistically significant at a level of statistical significance α = 0.05 (intercept *p*-value = 2.68 × 10^−12^; slope *p*-value = 1.25 × 10^−6^). The residuals distribution is characterized by a constant variance pattern, which confirms a good quality of the regression model. Variance of residuals is normally distributed, which confirms the correctness of the assumptions made for the regression model.

**Figure 11:**
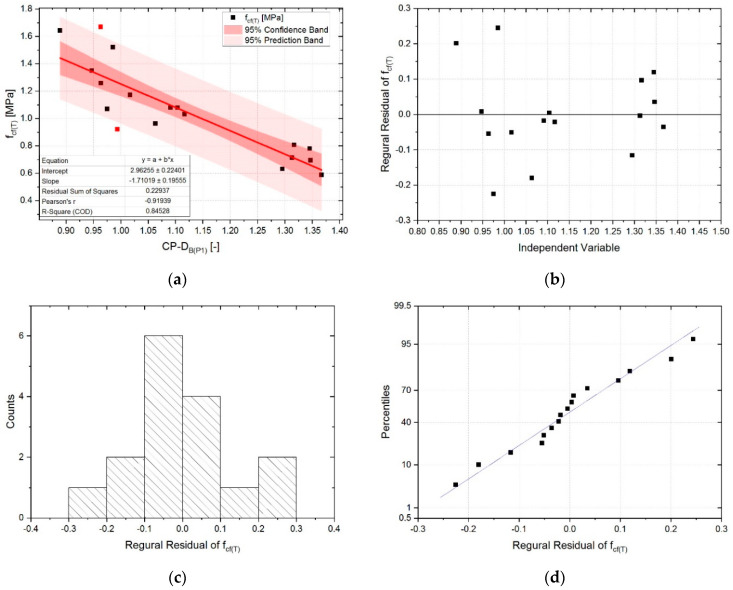
Results of the linear regression analysis for the *CP-D_B(P1)_-f_cf(T)_* relationship together with residual plots: (**a**) regression line (red points—outliers); (**b**) residuals vs. independent; (**c**) histogram of residuals; (**d**) normal probability of residuals.

The results of linear regression analysis for *CP-D_B(P1)_-f_cf(T)_* dependence are presented in Figure 11. Two outliers were identified, which for the final position of the regression line are outside the 95% prediction band. Elimination of these two points from the analysis allowed the quality of the linear regression model to be increased as well as the value of Pearson correlation coefficient to be increased in the same way as for the *CD_(P1)_-f_cf(R)_* dependence, i.e., from −0.87 to −0.92. The quality of model fit to the empirical data, measured by the *R-Square* is very high and equal to 0.85. The residuals values are much smaller than for the *CD_(P1)_-f_cf(R)_*. This is mainly due to the fact that the thermal degradation of the lime cement matrix caused a decrease in *f_cf_*. The linear regression model and its coefficients are statistically significant (α = 0.05), for intercept *p*-value = 2.66 × 10^−9^, and for slope *p*-value = 4.78 × 10^−7^. Variance of residuals is characterized by a constant pattern. No increasing or decreasing trends were observed in this aspect. Analysis of the normality of variance indicates that the assumption regarding the normality of distribution is true. The above observations indicate that all assumptions to the model are correct.

Calculated regression lines for the *CD_(P1)_-f_cf(R)_* and *CP-D_B(P1)_-f_cf(T)_* allow very accurate estimation of the tensile strength on the basis of measuring the cracks characteristics of the lime cement matrix. The development of such equations is of great practical importance and contributes to the development of non-destructive testing methods in cement composites technology.

## 5. Summary and Conclusions

The paper describes an innovative approach to the analysis of the cracking patterns of lime cement matrix subjected to the thermal load. For this purpose, an image-processing method was used. The cracked surface of the cement matrix was scanned and then an original procedure of the image double-segmentation was developed, in which machine-learning algorithms were implemented to extract cracks on the surface. The cracking patterns, presented as a binary image, were subjected to quantitative analysis. Three parameters were defined and examined: *TCA*—total crack area, *CD*—crack density, and *CP-D_B_*—fractal dimension of the cracking pattern. The application of the fractal dimension in cement composites technology is already known, however, the studies described in the literature to date indicate the use of this parameter for the study of morphology of the fracture zones created as a result of material destruction. In this paper, the fractal dimension has been used to assess the complexity of the cracking patterns, which has been unprecedented in this research aspect so far. Computer image analysis was conducted using the open-source *ImagejJ* software together with a machine-learning tool—the Trainable Weka Segmentation.

The results obtained indicate a very high usefulness of the fractal dimension to describe the complexity of the cracking patterns. An unquestionable advantage of *CP-D_B_* in comparison to *TCA* and *CD* are very small values of the variation coefficients. This property allows even small but statistically significant changes in the structure of the cracking patterns to be detected. The values of *CP-D_B_*, *TCA* and *CD* increase as the amount of cement in the cement matrix decreases and also increase with the thermal load of the material. The application of the two-phase thermal load has provided information on how the structure of the cracking patterns develops on the surface of the lime cement matrix.

Modification of the lime cement matrix with MS allowed the influence of this additive on the structure of the cracking pattern to be assessed. While the use of the pozzolanic additive increased the compressive strength of the lime cement matrix, the tensile strength was reduced. This is undoubtedly related to the much more complex structure of the cracking pattern of the CP32.5 + M series compared to the CP32.5. All three parameters, i.e., *CP-D_B_*, *TCA*, and *CD* were characterized by much higher values. This is mainly due to the increased brittleness of the lime cement matrix when modified with MS. Thus, while the most important functional feature of the cement matrix has been improved, i.e., *f_c_*, the more developed cracks structure favors progressive degradation of the material and reduction of its durability. The analysis also showed a very high correlation between *CP-D_B_* and two other parameters describing the cracking patterns, i.e., *TCA* and *CD*. The correlation coefficients were equal to 0.92 and 0.96, respectively.

The influence of the *w/c* ratio on the mechanical parameters and the cracking patterns is very clear. In the case of mechanical properties, together with increasing the concentration of cement grains in the cement matrix, there is a significant increase in compressive and tensile strength. The situation is similar in the case of reference samples and samples subjected to the thermal load. The results obtained confirm the commonly known trend. Bearing in mind the characteristics of the cracking patterns—*CP-D_B_*, *TCA*, and *CD*—the values of all three parameters increase with increasing the *w/c*. Thus, there are more cracks, they are more densely packed on the surface of the material, and their structure is more developed in a situation when the concentration of cement grains in the volume of the lime cement matrix decreases.

The basic mechanical properties of the lime cement matrix were also examined, both for reference samples and for those loaded with the elevated temperature. Compressive strength—*f_c_*, tensile strength—*f_cf_*, and brittleness, expressed as *f_cf_/f_c_* ratio, were determined. Correlations between mechanical properties and parameters of the cracking patterns were checked for estimation purposes. The relationships with *f_c_* were the least correlated, in this case the correlation strength was mostly weak or low. The correlations with *f_cf_/f_c_* were characterized by moderate, high or very strength. However, the relationships with *f_cf_* were best correlated. Correlation coefficients had very high negative values from −0.80 to −0.89. This indicates that the degree of complexity of the surface cracks structure to a very large extent influences the tensile strength of the lime cement matrix. A linear regression analysis was also performed for the two strongest relationships, i.e., *CD_(P1)_-f_cf(R)_* and *CP-DB_(P1)_-f_cf(T)_*, which allowed to define very high quality regression equations. The possibility of estimating mechanical properties on the basis of the non-destructive characterization of the cracking patterns is very practical and indicates the development of non-destructive testing methods in the cement composites technology.

## Figures and Tables

**Table 1:** Mechanical properties of the lime cement matrix, both for reference samples and those subjected to the thermal load.

Series	*w*/*c*	*f_c(R)_* [MPa]	*f_c(T)_* [MPa]	Difference between *f_c(R)_* and *f_c(T)_* [%]	*f_cf(R)_* [MPa]	*f_cf(T)_* [MPa]	Difference between *f_cf(R)_* and *f_cf(T)_* [%]	*f_cf(R)_/f_c(R)_*	*f_cf(T)_/f_c(T)_*	Difference between *f_cf(R)_/f_c(R)_* and *f_cf(T)_/f_c(T)_* [%]
CP32.5	0.45	56.34	35.86	−36.3	5.41	1.61	−70.2	0.096	0.045	−53.2
	0.50	45.50	28.07	−38.3	4.82	1.23	−74.6	0.106	0.044	−58.8
	0.55	37.66	17.36	−53.9	4.17	1.02	−75.6	0.111	0.059	−47.0
CP32.5 + M	0.45	64.40	42.53	−34.0	4.38	1.06	−75.8	0.068	0.025	−63.3
	0.50	56.90	39.61	−30.4	3.63	0.74	−79.6	0.064	0.019	−70.7
	0.55	49.24	28.78	−41.6	2.60	0.66	−74.4	0.053	0.023	−56.2

**Table 2:** Values of coefficients of variation for the results of mechanical parameters.

Series	*w/c*	*f_c(R)_* [%]	*f_c(T)_* [%]	*f_cf(R)_* [%]	*f_cf(T)_* [%]
CP32.5	0.45	3.0	3.0	8.7	4.0
	0.50	4.3	9.4	8.1	9.6
	0.55	6.4	8.8	8.5	10.8
CP32.5 + M	0.45	6.6	5.4	8.8	2.1
	0.50	2.2	4.0	3.9	10.5
	0.55	5.8	6.0	9.4	8.2

**Table 3:** Changes in the characteristics of cracks between the first and second phase of the thermal load.

Series	*w/c*	Difference in *TCA*, *CD*, and *CP-D_B_* after Second Phase of the Thermal Load with Respect to the First Phase [%]		
		*TCA*	*CD*	*CP-D_B_*
CP32.5	0.45	+104.7	+27.8	+11.1
	0.50	+84.5	+18.4	+9.9
	0.55	+94.1	+25.5	+11.2
CP32.5 + M	0.45	+70.4	+20.2	+6.3
	0.50	+68.1	+21.9	+5.4
	0.55	+62.2	+11.7	+4.1

**Table 4:** Matrix of correlation coefficients between the features describing the cracking patterns and the mechanical properties of the lime cement matrix.

	*Pearson Correlations (r)*						*Spearman Correlations (ρ)*					
	*f_c(R)_*	*f_c(T)_*	*f_cf(R)_*	*f_cf(T)_*	*f_cf(R)_/f_c(R)_*	*f_cf(T)_/f_c(T)_*	*f_c(R)_*	*f_c(T)_*	*f_cf(R)_*	*f_cf(T)_*	*f_cf(R)_/f_c(R)_*	*f_cf(T)_/f_c(T)_*
*TCA_(P1)_*	0.25	0.27	−0.82 *	−0.81 *	−0.87 *	−0.78 *	0.25	0.25	−0.80 *	−0.82 *	−0.78 *	−0.72 *
*TCA_(P2)_*	0.24	0.26	−0.83 *	−0.81 *	−0.86 *	−0.77 *	0.22	0.19	−0.82 *	−0.87 *	−0.75 *	−0.69 *
*CD_(P1)_*	0.04	0.06	−0.87 *	−0.84 *	−0.78 *	−0.65 *	0.04	0.01	−0.88 *	−0.87 *	−0.68 *	−0.56 *
*CD_(P2)_*	0.05	0.08	−0.85 *	−0.84 *	−0.76 *	−0.65 *	0.06	0.01	−0.86 *	−0.87 *	−0.65 *	−0.54 *
*CP-D_B(P1)_*	0.20	0.24	−0.83 *	−0.87 *	−0.83 *	−0.77 *	0.22	0.24	−0.85 *	−0.88 *	−0.79 *	−0.73 *
*CP-D_B(P2)_*	0.16	0.18	−0.83 *	−0.86 *	−0.80 *	−0.71 *	0.15	0.13	−0.85 *	−0.89 *	−0.69 *	−0.65 *

* Correlation is significant at the 0.05 level. Designation of correlation strength: |r|<0.2—weak correlation; |r|∈〈0.2÷0.4)—low correlation; |r|∈〈0.4÷0.6)—moderate correlation; |r|∈〈0.6÷0.8)—high correlation; |r|∈〈0.8÷0.9)—very high correlation; |r|∈〈0.9−1.0〉—correlation virtually complete
